# Entomopathogenic Nematode *Steinernema rarum* and its Symbiotic Bacterium against Fire Ants (*Solenopsis* sp.) under Laboratory Conditions

**DOI:** 10.1007/s13744-026-01400-y

**Published:** 2026-05-21

**Authors:** Carolina Egidio Babesco, Julie Giovanna Chacón-Orozco, Luís Garrigós Leite, Ricardo Harakava, Ana Eugênia de Carvalho Campos

**Affiliations:** 1 Laboratorial de Referência Em Pragas Urbanas, Unidade, Instituto Biológico, São Paulo, SP Brazil; 2 Serviço Laboratorial de Referência Em Controle Biológico, SP, BR, Instituto Biológico, Campinas, SP Brazil; 3 Serviço Laboratorial de Referência Em Biologia Molecular Aplicada, Instituto Biológico, São Paulo, SP Brazil

**Keywords:** Biological control, *Xenorhabdus szentirmaii*, Secondary metabolites, Insect pest, Fire ant

## Abstract

The genus *Solenopsis* comprises approximately 191 species, commonly known as fire ants. These ants are highly aggressive when the colony is disturbed and can cause accidents due to their stings. In addition, they cause economic losses in agriculture by hindering manual harvesting, feeding on crops, and establishing mutualistic relationships with sap-sucking insects. Therefore, there is considerable interest in the development of new and effective methods and products for the control of these insects. The aim of this study was to evaluate the activity of the entomopathogenic nematode *Steinernema rarum* and its symbiotic bacterium against fire ants (*Solenopsis invicta*) under laboratory conditions. To assess nematode activity at different concentrations (5, 50, 150, and 300 IJs per insect), as well as the effects of the symbiotic bacterium of *S. rarum* and its secondary metabolites (SM), each treatment was applied separately to different ant developmental stages (larvae, pupae, workers, and winged forms) maintained in Petri dishes. In addition, a microcolony experiment was conducted, consisting of ten workers, five larvae, five pupae, and one winged form per dish. The results demonstrated that all treatments with nematodes, the bacterium, and its metabolites caused mortality in ants, ranging from 10 to 100% depending on the developmental stage and treatment. These findings highlight the potential of entomopathogenic nematodes and their symbiotic bacteria for the control of fire ants. However, further studies are required to develop suitable formulations and management strategies for future field applications.

**Supplementary Information:**

The online version contains supplementary material available at 10.1007/s13744-026-01400-y.

## Introduction

Ants are social insects of the family Formicidae and are characterized by their wide distribution, abundance, and species richness in terrestrial ecosystems (Donat et al. 2000). All known species are eusocial, characterized by overlapping generations, sterile and reproductive castes, and cooperative brood care (Wilson 1971). Due to their high adaptive capacity, ants occur abundantly even in anthropogenically disturbed environments (Martins et al. 2021). Once established in areas with close human interaction, some ant species may cause economic damage and pose risks to public health, either as vectors of pathogens or due to the venom delivered through stings inflicted by certain species (Ramalho et al. 2022).

The genus *Solenopsis* originated in South America and comprises 191 valid species and 21 subspecies (ANTWEB 2025), of which 161 occur in the Neotropical Region (Baccaro et al. 2015). These include the ants known in Brazil as fire ants (“formigas lava pés”). Fire ants of the *saevissima* species complex include ecologically and economically important species such as *S. invicta*, *S. saevissima*, and *S. richteri* (Pitts, 2002). *Solenopsis invicta* Buren is one of the world’s most important invasive species, widely distributed in South America and introduced into several continents (Buren 1972). *S. richteri* has a more restricted distribution in the Southern Cone, whereas *S. saevissima* is widely distributed in Brazil, especially along the coast and in the Amazon, where it is considered a pest, although no invasions outside the country have been reported (Trager 1991).

These ants are highly aggressive when colonies are disturbed and during foraging, and their stings can cause effects ranging from localized pain to anaphylactic shock in allergic individuals due to their venom (Martins, 2010). Fire ants are omnivorous and opportunistic, feeding on a wide variety of plant and animal material and household foods (Campos et al. 2017). They also feed on honeydew excreted by sap-sucking insects, which further exacerbates their impact in agricultural systems (Morales and Beal 2006).

Some fire ant species were accidentally transported from South America to other regions of the world, particularly to the United States, via ships carrying timber (Pitts et al. 2005). Among these, *S. invicta* is responsible for the most severe problems in all regions where it has established, causing serious health issues and substantial agricultural losses, and is currently considered one of the most important invasive insects worldwide (Henshaw et al. 2005). Consequently, there is strong interest in the development of new products and methods that are effective for controlling these insects.

Entomopathogenic nematodes (EPNs) of the genera *Steinernema* and *Heterorhabditis* are considered among the most successful biological tools used for the control of soil-dwelling pests. They possess nearly all the attributes of an ideal biological control agent. These nematodes enter the host through natural openings such as the mouth, anus, or spiracles, or, in some cases, directly penetrate the insect cuticle (e.g., *Heterorhabditis* spp.) (Grewal et al. 2005). Once in the hemocoel, they release symbiotic bacteria (*Xenorhabdus* or *Photorhabdus*), which are responsible for insect virulence, causing death by septicemia within up to 48 h (Tarasco et al. 2023).

These organisms present several advantages that make them effective biological control agents, including adaptability, capacity to colonize soil as inundative organisms, host-seeking behavior against soil-dwelling pests, high virulence, safety for vertebrates and plants, ease of mass production under laboratory conditions, compatibility with conventional application equipment, and compatibility with chemical and biological pesticides (Leite et al. 2004).

Therefore, the objective of this study was to evaluate the action of the entomopathogenic nematode *Steinernema rarum*, its symbiotic bacterium (*Xenorhabdus szentirmaii*), and its secondary metabolites for the control of fire ants under laboratory conditions.

## Materials and methods

### Collection of fire ant colonies in the field and laboratory maintenance

*Solenopsis* colonies were collected at the Instituto Biológico, São Paulo, using shovels, transferred to plastic buckets, and transported to the laboratory. Ants were separated from the soil by slow flooding with water using an adapted IV infusion set, forcing workers, queens, and brood to move to the surface and into a wooden collection box.

Colonies were maintained in polyethylene trays coated with petroleum jelly (1:1) to prevent escape. Petri dishes lined with plaster and covered with red cellophane served as artificial nests. Maintenance included continuous provision of water, sugar solution, and larvae of *Tenebrio molitor* Linnaeus, 1758 (Coleoptera: Tenebrionidae).

### Entomopathogenic nematode and symbiotic bacterium

The entomopathogenic nematode (EPN) *Steinernema rarum* PAM25 is deposited in the entomopathogenic microorganism collection of the Biological Control Reference Laboratory Service, Instituto Biológico (DAPSA–Campinas, SP), and was selected due to its availability for large-scale production (biphasic fermentation: liquid and solid). Bacterial isolation (*Xenorhabdus szentirmaii*) was performed from the hemolymph of infected hosts following Akhurst (1980). *Galleria mellonella* Linnaeus, 1758 (Lepidoptera: Pyralidae) larvae were inoculated with 1 mL of a suspension containing *S. rarum* PAM25. After insect death, larvae were surface-sterilized with 90% ethanol and flame-sterilized. Hemolymph was collected using an entomological pin and plated on NBTA medium. Plates were incubated in the dark for 72 h at 27°C. Blue bacterial colonies were subcultured until uniform morphology and primary (phase I) form were obtained.

### Liquid fermentation and extraction of secondary metabolites

A colony of *X. szentirmaii* was cultured in 25% LB medium due to the toxicity of full-strength medium previously observed in workers (Babesco et al. 2025) and incubated under constant agitation for 144 h at 25°C (bacterial treatment). For the cell-free secondary metabolite treatment (SM), the bacterial suspension was autoclaved at 121°C for 15 min.

### Evaluation of mortality of brood and adults of fire ants inoculated with Steinernema rarum PAM25 and its symbiotic bacterium

Brood and adults were exposed to EPN treatments at different concentrations (5, 50, 150, and 300 IJs per individual), to *X. szentirmaii*, and to its secondary metabolites (SM). Each experimental unit consisted of a Petri dish (9 cm diameter) lined with filter paper (Whatman No. 1), containing 1.5 mL of treatment and ten ants evaluated separately by developmental stage (larvae, pupae, workers, and winged forms), following a methodology adapted from Glazer and Lewis (2000). Sterile distilled water and LB 25% medium were used as controls. Each treatment had nine replicates. Mortality and visible symptoms, particularly changes in ant coloration, were evaluated on the seventh day after inoculation. For ants exposed to nematodes, the White trap method (White 1927) was used to isolate and confirm nematode presence.

### Evaluation of mortality of red imported fire ants in microcolonies inoculated with the nematode *Steinernema rarum*, the symbiotic bacterium, and its metabolites

Microcolonies were exposed to EPN treatments at different concentrations (5, 50, 150, and 300 IJs per individual), bacterium, secondary metabolites (SM), bacterial medium, and water (control). Each experimental unit consisted of a 9-cm Petri dish lined with filter paper (Whatman No. 1), containing 1.5 mL of treatment and a microcolony composed of ten workers, five pupae, five larvae, and one winged form. Each treatment had six replicates. Mortality and visible symptoms, particularly changes in ant coloration, were evaluated on the seventh day after inoculation. For ants exposed to nematodes, the White trap method (White 1927) was used to isolate and confirm nematode presence.

### Identification of collected specimens

Ant identification followed the proposal of Hebert et al. (2003), using mitochondrial DNA, specifically the COI (cytochrome oxidase I) gene. Partial COI sequences were generated from specimens collected from multiple nests used in the laboratory assays and compared with sequences deposited in the GenBank database using the BLAST application. (NCBI). Species identification was confirmed when sequence similarity exceeded 99%, with high score values and E-values of zero or close to zero (BLAST 2022).

### Statistical analysis

Mortality percentage data were corrected relative to the control and transformed using the equation √(X/100), where X represents the mortality value of each treatment replicate. The data were then subjected to one-way analysis of variance (ANOVA), followed by Tukey’s multiple comparison test at a 5% significance level to compare treatment means. For each analysis, the exact p-values, F statistics, and corresponding degrees of freedom (df) were calculated and reported. Mortality distributions among treatments were graphically represented using boxplots. For the nematode assays conducted with isolated developmental stages, dose–response analysis was performed to estimate the lethal doses required to cause 50% and 90% mortality (LD₅₀ and LD₉₀), together with their 95% confidence intervals. All statistical analyses and graphical representations were performed using the R software (R Core Team 2025). The complete statistical outputs, η^2^ estimates with their 95% confidence intervals, are provided in the Supplementary Material.

## Results and discussion

### Evaluation of mortality of brood and adults of fire ants inoculated with the entomopathogenic nematode *Steinernema rarum*

**Fig. 1:**
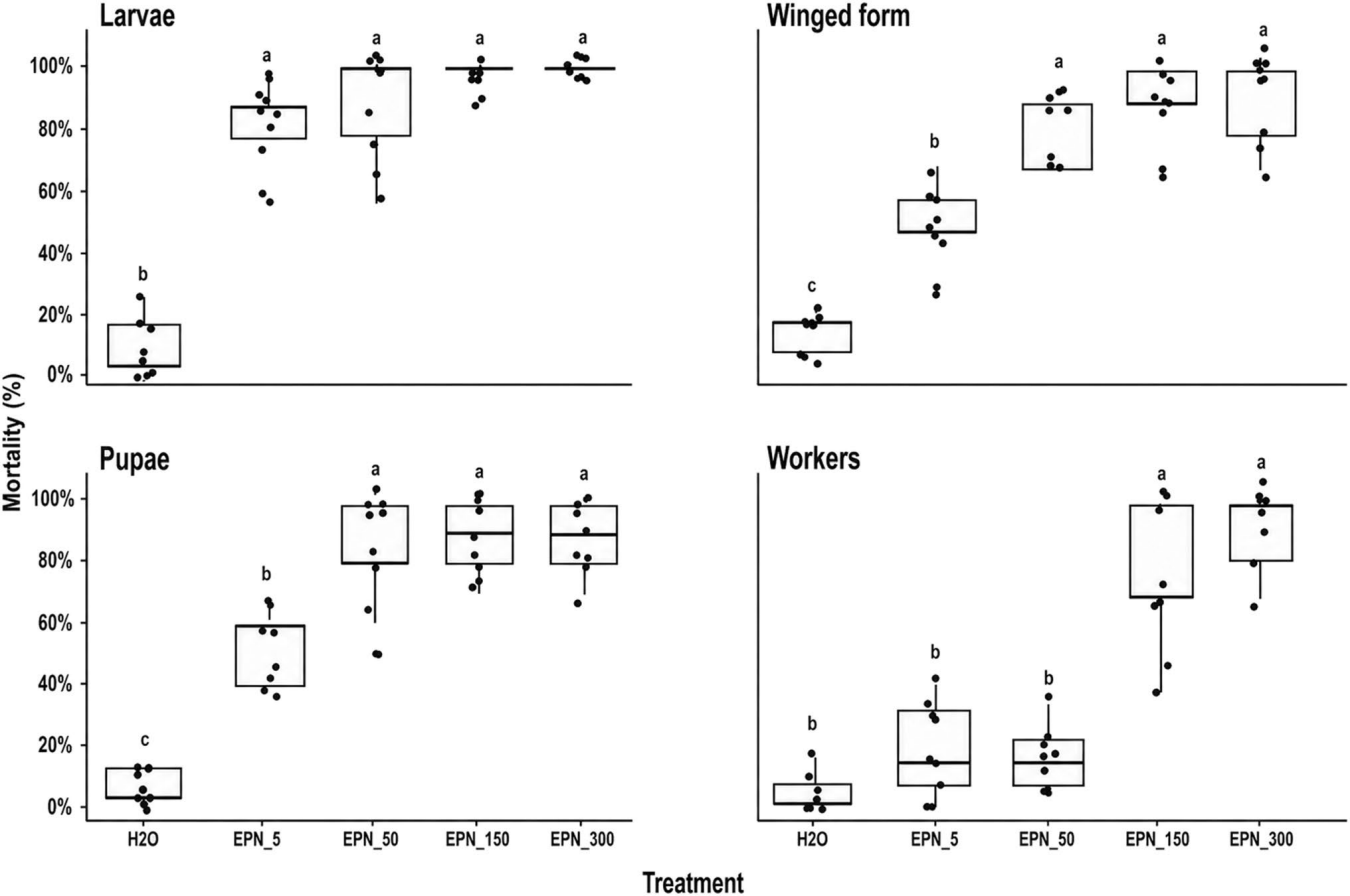
Mortality (%) of larvae, pupae, winged forms, and workers of ants exposed to different concentrations of EPNS and the control treatment (H₂O), evaluated seven days after inoculation. Boxplots show median, interquartile range, and dispersion of replicates. Different letters indicate significant differences among treatments within each developmental stage or caste (Tukey, *P* < 0.05)

The treatments significantly affected mortality in all evaluated stages. For larvae, a strong treatment effect was observed (F = 58.03; df = 4,40; *p* < 0.001), and all EPNs concentrations resulted in high mortality (86.7–100%), with no significant differences among concentrations, but all differed from the water control according to Tukey’s test (Fig. 1). For pupae (F = 121.10; df = 4,40; *p* < 0.001) and winged forms (F = 47.80; df = 4,40; *p* < 0.001), mortality increased with EPN concentration, with EPN 50, 150, and 300 IJ/insect showing significantly higher mortality than the control and the lowest concentration treatment. For workers (F = 34.03; df = 4,40; *p* < 0.001), the highest concentrations (EPN 150 IJ/insect and EPN 300 IJ/insect) caused significantly greater mortality, whereas lower concentrations did not differ from the control, indicating greater tolerance in this caste. The consistently high effect sizes observed across all stages indicate a strong influence of treatment on mortality. Dose–response analysis further supported differences in susceptibility among stages, with larvae showing the lowest LD₅₀ and LD₉₀ values, indicating high sensitivity, whereas workers presented the highest lethal dose estimates, consistent with greater resistance to the nematode treatment. Pupae and winged forms showed intermediate susceptibility (Table 1).

**Table 1:** Estimated LD₅₀ and LD₉₀ values (95% confidence intervals) for different developmental stages and castes of ants exposed to EPNS

STAGE	LD₅₀ (95% CI)	LD₉₀ (95% CI)
Larvae	0.05 (− 0.16 to 0.27)	13.79 (− 4.40 to 31.98)
Pupae	1.65 (− 0.69 to 3.99)	278.51 (− 162.91 to 719.93)
Workers	89.93 (66.32 to 113.53)	277.07 (109.07 to 445.06)
Winged forms	2.75 (0.30 to 5.19)	166.73 (− 28.66 to 362.13)

Negative lower confidence interval values reflect the statistical uncertainty of the dose–response model and should not be interpreted as biologically meaningful doses

**Fig. 2:**
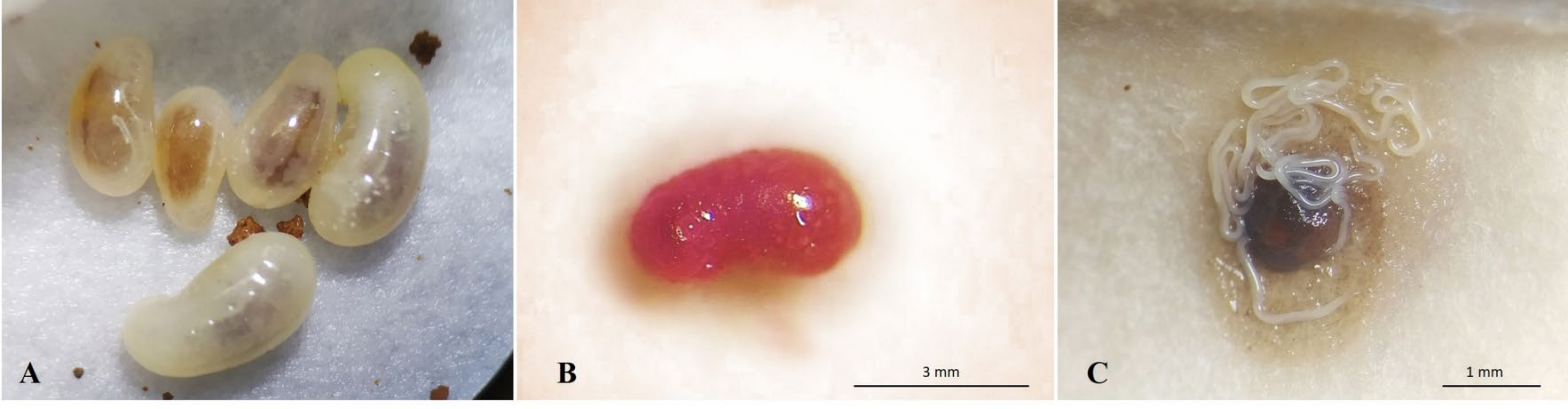
**A**) Healthy larvae of *Solenopsis invicta*; **B**) larva infected with *Steinernema rarum* (4 days after inoculation); **C**) EPNs emerging from an infected larva (5 days after inoculation)

**Fig. 3:**
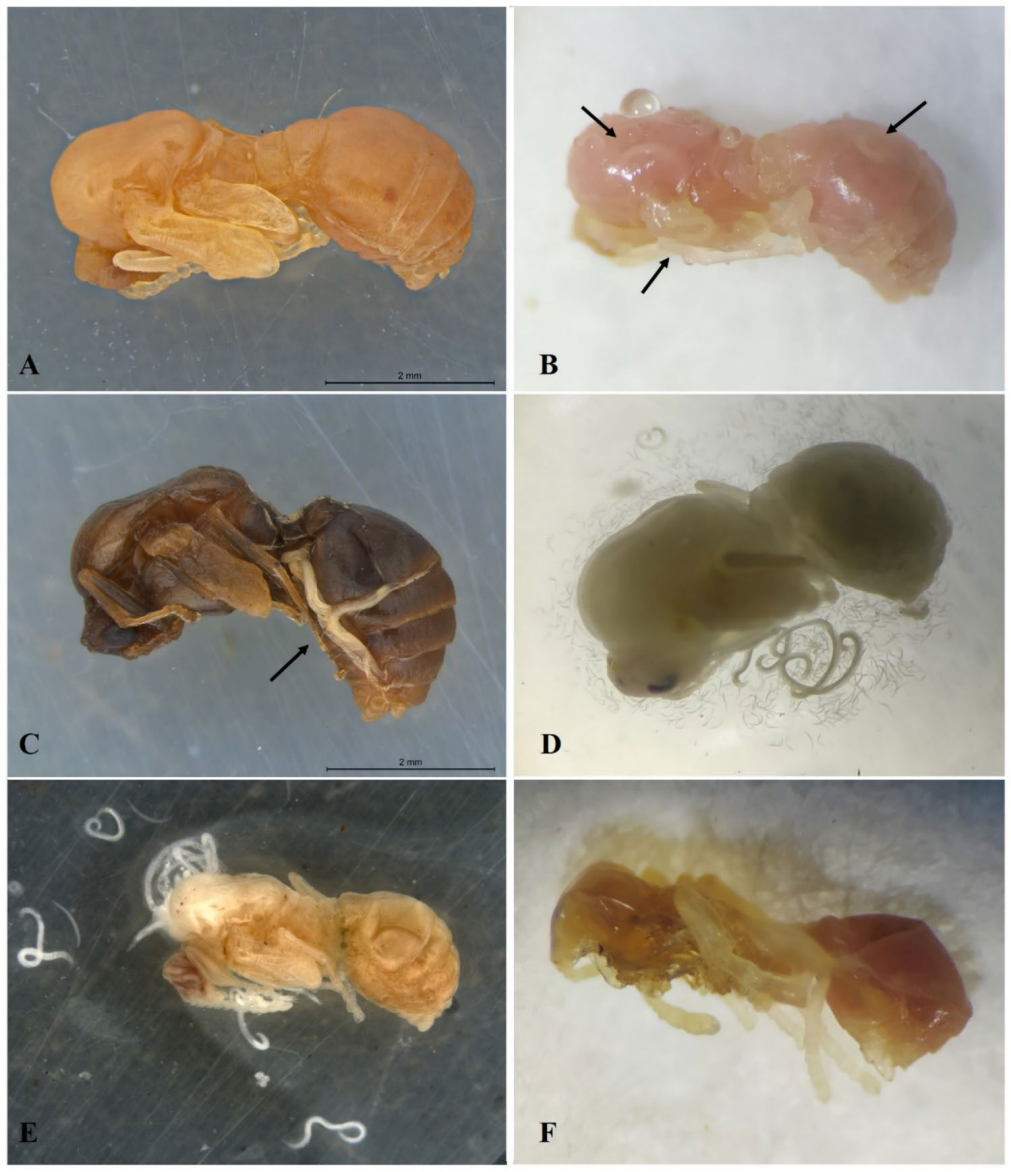
(**A**) Healthy pupa of *Solenopsis invicta*; (**B**) Dead pupa (3 days post-inoculation) showing reddish coloration due to the release of symbiotic bacteria, with developing adult entomopathogenic nematodes (EPNs) inside the pupa (arrow indicates); (**C**) Adult EPNs developing inside the pupa; (**D**) Infective juveniles and adults emerging from the infected pupa (5 days post-inoculation); (**E**) Adult EPNs emerging through openings left by infective juveniles (6 days post-inoculation); (**F**) Dried pupa after EPN emergence (7 days post-inoculation)

High susceptibility of the brood to nematode infection was observed, as evidenced by the high mortality rates, probably due to their immobility and lack of effective defense mechanisms. From the third day after EPN application, larvae and pupae began to exhibit a pinkish to reddish coloration, which is characteristic of infection caused by this nematode species (Figs. 2 and 3). This color change appears to be exclusive to *S. rarum* and has not been reported for other *Steinernema* species. Nguyen et al. (2006) also observed this coloration change in *Galleria mellonella* larvae inoculated with *S. rarum* three days after infection.

In larvae that died and showed EPN emergence, only the gut remained intact (Fig. 2C). This likely occurred due to the presence of gut microorganisms antagonistic to the bacterium *Xenorhabdus szentirmaii*, which may have prevented tissue degradation and, consequently, digestion by the EPNs developing inside the larva. Zhukova et al. (2017) identified Mollicutes (parasites living on or within host cells), as well as *Pseudomonas* and *Enterobacter*, in the gut of *Atta* ants. These bacteria are also found in fungal gardens but are rarely, if ever, detected in adult workers, suggesting that they perform specific beneficial functions related to larval growth and development. Similarly, Xiao et al. (2023) identified gut symbionts in *S. invicta*, with Proteobacteria as the dominant group. The authors emphasized that these bacteria are closely associated with insect development, nutrient metabolism, and disease resistance, although the main factors modulating these communities remain incompletely understood.

In dead pupae, a reddish coloration was also observed (Fig. 3B), and nematode emergence began on the fifth day after inoculation (Fig. 3D). After the complete emergence of infective juveniles, the pupae exhibited a dry and dehydrated appearance (Fig. 3F).

**Fig. 4:**
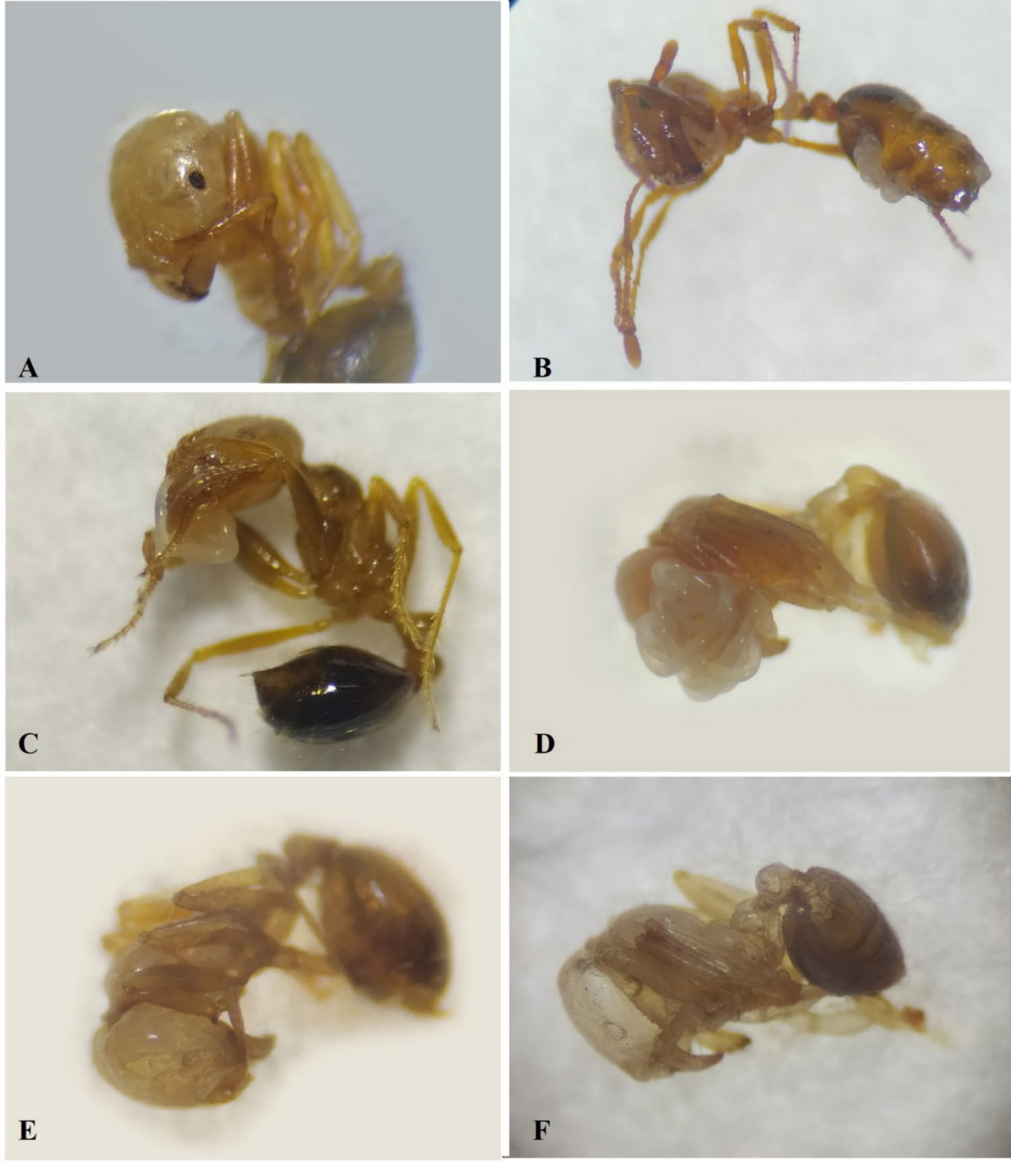
(**A**) Workers infected by *Steinernema rarum* in the head region (4 days post-inoculation); (**B**) Entomopathogenic nematodes (EPNs) emerging from the abdomen; (**C**) EPNs emerging from the mouthparts (6 days post-inoculation); (**E**) Juveniles feeding on the head region; (**F**) Worker carcass after the emergence of adults and infective juveniles (8 days post-inoculation)

Workers parasitized by nematodes consistently died in a bent posture, with nematode emergence observed (Fig. 4), although this occurred less frequently than in brood and winged forms.

Wu et al. (2023) tested seven nematode species against *Solenopsis invicta*. In their experiment, 30 workers and 20 larvae were added to containers containing 250 g of autoclaved soil, and 2 mL of a suspension containing 10,000 IJs mL⁻^1^ was applied. Among workers, *Heterorhabditis bacteriophora* caused the highest mortality rate (56%), whereas for larvae, *Steinernema longicaudum* resulted in 98% mortality. In the same study, the authors conducted an experiment using *Galleria mellonella* larvae infected with 200 IJs of *S. riobrave* or *H. bacteriophora* to evaluate ant preference between frozen larvae and infected larvae. All workers showed a preference for frozen larvae and avoided infected ones, indicating that ants are likely able to detect the presence of nematodes.

**Fig. 5:**
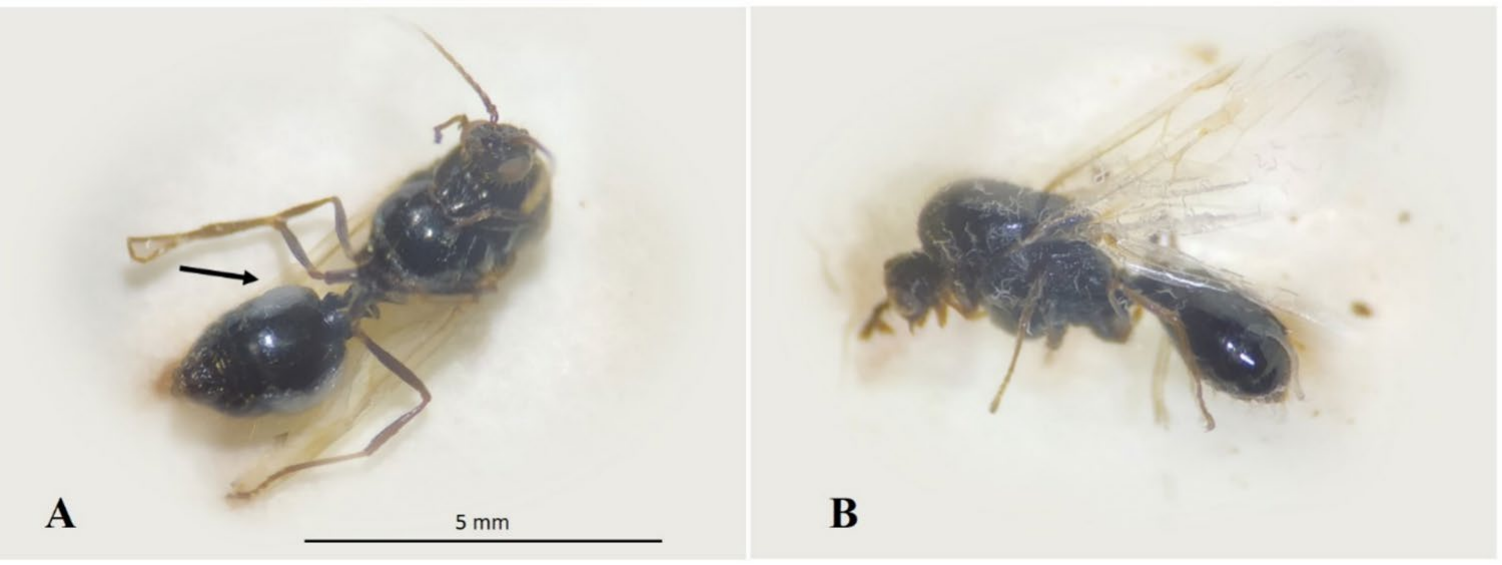
(**A**) Dead winged form in an inverted dorsal position, with entomopathogenic nematodes (EPNs) emerging from the abdomen (arrows indicate); (**B**) Infective juveniles emerging from the thoracic region

Winged forms were more sensitive to the treatments and generally died more rapidly than workers, typically in an inverted dorsal position (Fig. 5A). In these individuals, nematode emergence was also observed (Fig. 5B).

### Evaluation of mortality of brood and adults of fire ants inoculated with *Xenorhabdus szentirmaii* and its secondary metabolites

The treatments significantly affected mortality in larvae, pupae, and workers, as confirmed by ANOVA, whereas no significant effect was observed for winged forms (F = 1.40; df = 3,32; *p* = 0.262).

**Fig. 6:**
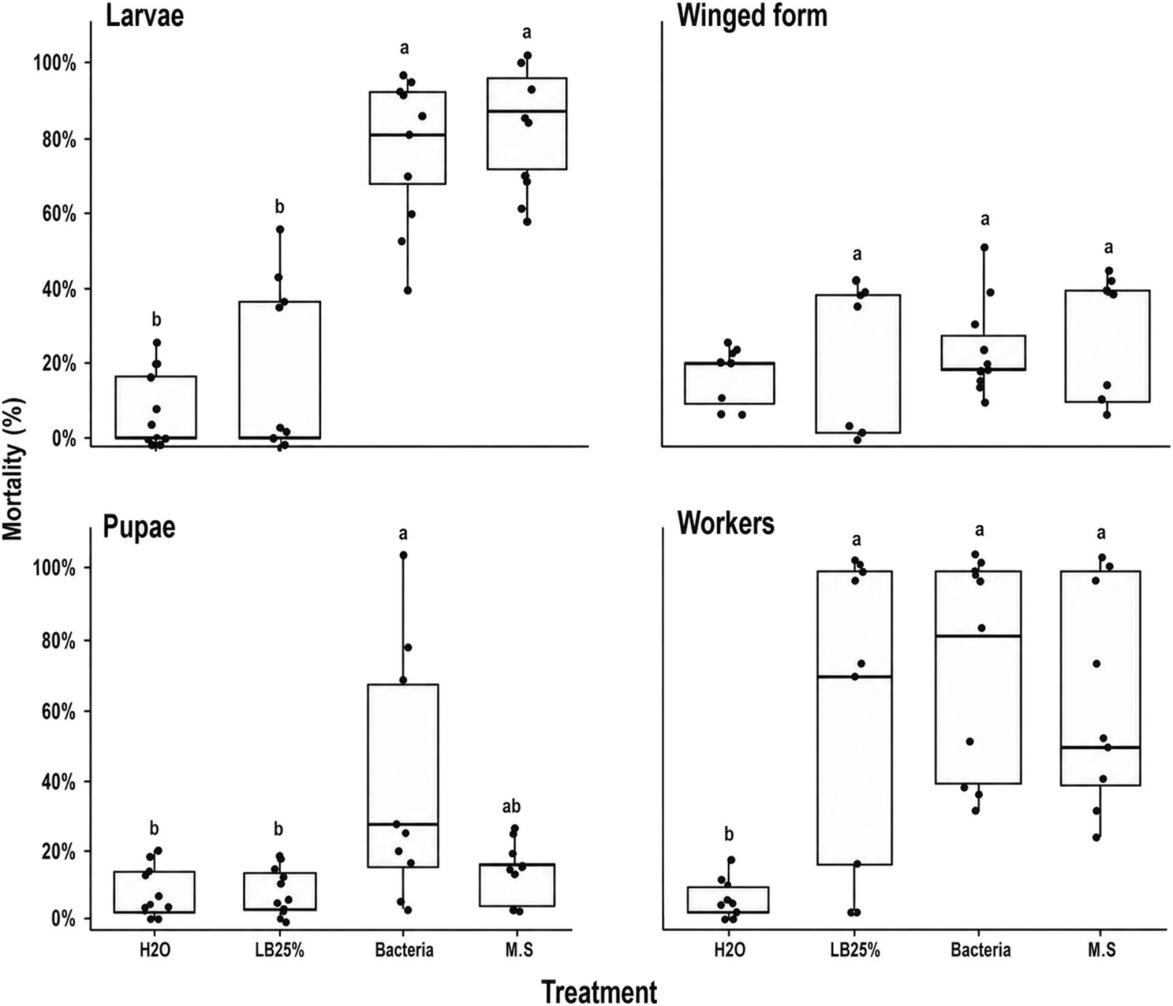
Mortality (%) of ant developmental stages and castes (larvae, pupae, winged forms, and workers) subjected to different treatments (H₂O, LB25%, Bacteria, and M.S.) seven days after inoculation. Boxplots represent the distribution of mortality values, with points indicating individual replicates. Different lowercase letters above the boxplots indicate significant differences among treatments within each group according to Tukey’s multiple comparison test (*P* < 0.05)

For larvae, a strong treatment effect was observed (F = 42.60; df = 3,32; *p* < 0.001), with treatments containing bacteria (75.6%) and secondary metabolites (85.6%) resulting in the highest mortality rates. These treatments did not differ significantly from each other, but both were significantly higher than the control (H₂O: 8.9%) and the LB25% treatment (16.7%) (Fig. 6).

For pupae, a significant effect was also detected (F = 4.98; df = 3,32; *p* < 0.01), with the bacterial treatment resulting in the highest mortality (28.9%), differing significantly from the control (3.3%) and LB25% (4.4%), whereas the secondary metabolites treatment showed intermediate values (8.9%).

In workers, treatments had a significant effect (F = 7.34; df = 3,32; *p* < 0.001), with bacteria (71.1%), secondary metabolites (62.2%), and LB25% (61.1%) showing similarly high mortality and not differing significantly from one another, all being superior to the control (7.8%).

For alates, no significant treatment effect was observed (F = 1.40; df = 3,32; *p* = 0.262), with mean mortality ranging from 1.67 (95% CI: 1.28–2.05) to 3.00 dead individuals, and no significant differences among treatments.

Overall, the results indicate high efficacy of bacterial and secondary metabolite treatments in larvae, moderate effects in pupae and workers, and limited efficacy in winged forms.

**Fig. 7:**
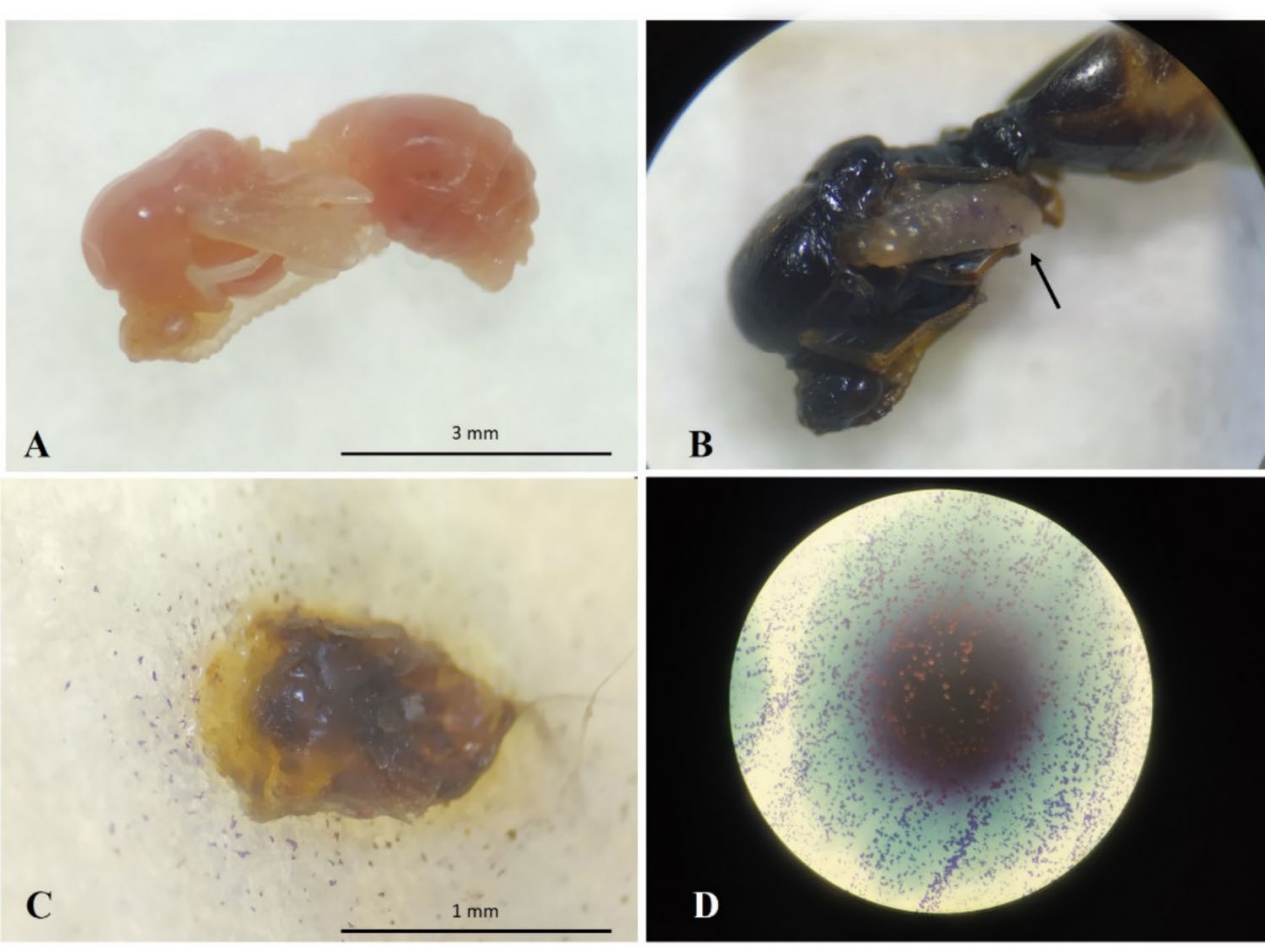
(**A**) Reddish pupa after application of secondary metabolites (4 days post-inoculation); (**B**) Dead pupa with formation of iodinin crystals on the wings (6 days post-inoculation); (**C**) Dead larva inoculated with the bacterium, showing iodinin crystals on the filter paper (4 days post-inoculation); (**D**) Iodinin crystals formed on the surface of *Xenorhabdus szentirmaii* colonies grown on NBTA medium

Symptoms of infection caused by the bacterium and its secondary metabolites were observed only in larvae and pupae and were evidenced by a color change to pinkish to reddish tones due to the presence of compounds produced by *Xenorhabdus szentirmaii* (Fig. 7A). The presence of Poly-Iodinin crystals (small purple spots) (Fodor et al. 2008), produced by the bacteria, was also observed inside larvae and pupae, as well as surrounding them on the filter paper (Fig. 7B and C). These crystals were also formed on the surface of bacterial colonies when cultured on NBTA medium (Fig. 7D). Seven days post-inoculation, the larvae were completely disintegrated.

Although the cell-free metabolite treatment provided indirect evidence that bacterial-derived compounds contribute to mortality, this approach does not fully replace a specific heat-killed bacterial control. Therefore, the relative contribution of inactive bacterial cell components versus secreted bioactive compounds could not be completely disentangled in the present study.

No studies evaluating the action of *Xenorhabdus szentirmaii* on ants were found. However, Orozco et al. (2018) demonstrated that secondary metabolites produced by *X. szentirmaii* have a strong inhibitory effect on the symbiotic fungus of leaf-cutting ants (*Leucoagaricus gongylophorus* (Möller, 1893) Singer, 1986 (Agaricales: Agaricaceae)), highlighting the potential of these compounds as an indirect biological control strategy for *Atta* colonies. de Paula et al. (2006) evaluated the pathogenicity of *Photorhabdus temperata* against the leaf-cutting ants *Acromyrmex subterraneus* (Forel, 1893) (Hymenoptera: Formicidae)and *Atta laevigata* (Smith, 1858) (Hymenoptera: Formicidae) through topical application of the bacterium to the gaster region of workers. The bacterium caused high mortality (> 80% and 60%, respectively) in both species within 24 h after inoculation.

Londoño et al. (2019) tested extracts from symbiotic bacteria of entomopathogenic nematodes (EPNs) against the leaf-cutting ant *Atta cephalotes* (Linnaeus, 1758) (Hymenoptera: Formicidae) and reported insecticidal activity by ingestion caused by *Photorhabdus* sp., as well as contact insecticidal activity caused by *X. nematophila*.

The application of bacteria for ant control may act through different mechanisms, including inhibition of the symbiotic fungus (*Leucoagaricus gongylophorus*) of leaf-cutting ants. Chacón-Orozco et al. (2018) demonstrated this effect by applying secondary metabolites of *Xenorhabdus szentirmaii*, which inhibited the development of the symbiotic fungus of *Atta sexdens*.

The use of symbiotic bacteria of EPNs has not yet been commercially explored; however, several studies have demonstrated their potential both for insect pest control and for disease management. There are reports of effectiveness against fungal pathogens (Chacón-Orozco et al. 2020; Kgosiemang et al. 2024) as well as against bacterial plant diseases (Chacón-Orozco et al. 2018; Ramos et al. 2023).

### Evaluation of mortality of red imported fire ants in microcolonies inoculated with the Nematode *Steinernema rarum*, the symbiotic bacterium, and its metabolites

The treatments (EPN) significantly affected colony mortality in the microcolonies, as confirmed by ANOVA.

**Fig. 8:**
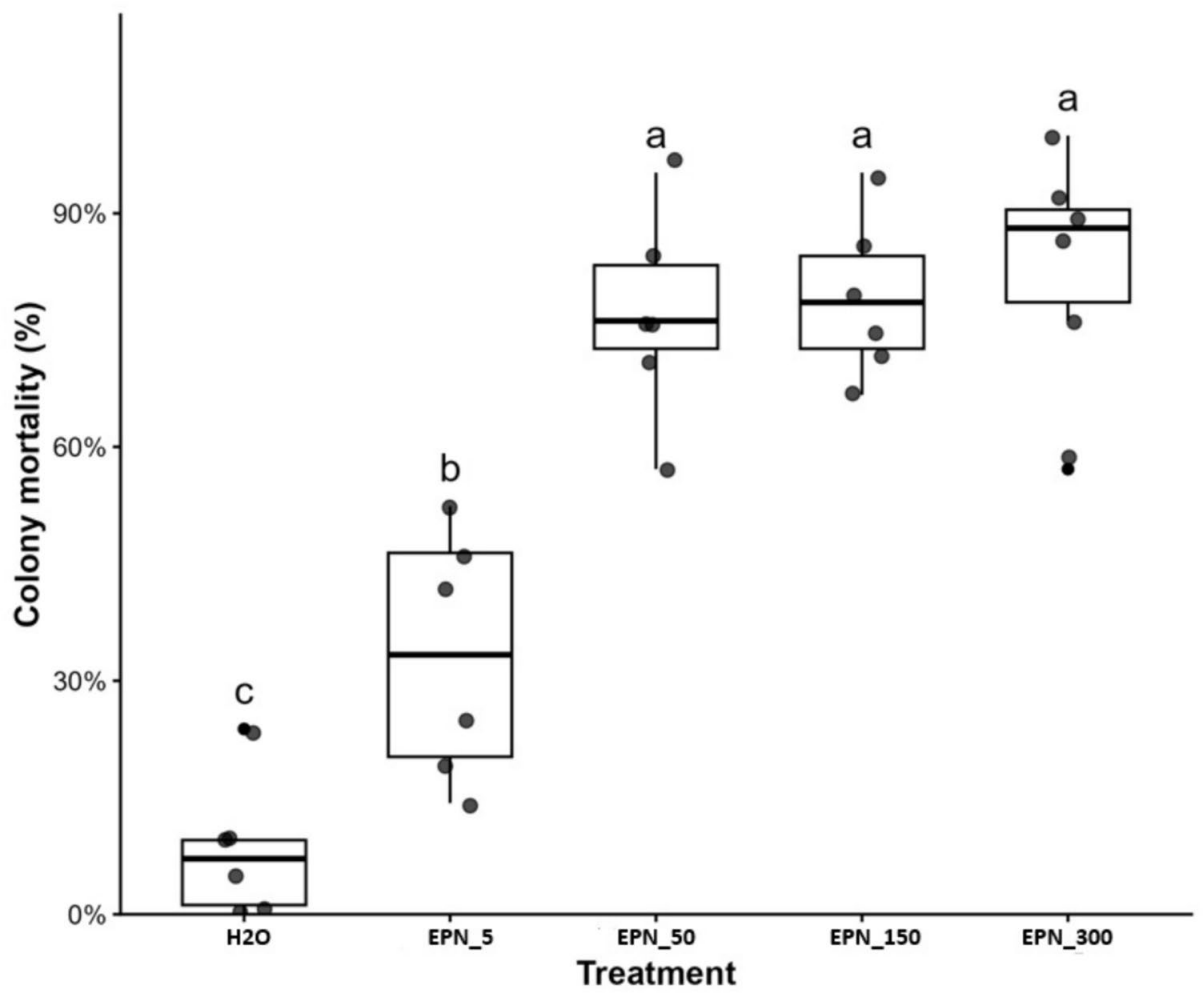
Colony mortality (%) of ant microcolonies exposed to increasing concentrations of EPNS and the water control (H₂O), evaluated seven days after inoculation. Boxplots show the median, interquartile range, and dispersion of replicates. Different lowercase letters indicate significant differences among treatments according to Tukey’s test (*P* < 0.05)

For larvae, a significant treatment effect was observed (F = 25.00; df = 4,25; *p* < 0.001). Colony mortality increased with increasing EPNS concentration, with the highest values observed for EPN_50, EPN_150, and EPN_300, which did not differ significantly from one another according to Tukey’s test (Fig. 8). These treatments resulted in mortality rates above 75%, whereas EPNS_5 showed intermediate mortality and the water control presented the lowest mortality.

For pupae, a significant treatment effect was also detected (F = 17.96; df = 4,25; *p* < 0.001). Mortality followed a similar pattern, with higher values in EPN_50, EPN_150, and EPN_300, intermediate mortality in EPN_5, and the lowest mortality in the water control.

In workers, treatments had a significant effect (F = 11.94; df = 4,25; *p* < 0.001). The highest concentrations (EPN_150 and EPN_300) resulted in greater mortality, followed by intermediate effects in EPN_50, while EPN_5 and the control showed lower mortality.

For alates, a significant treatment effect was also observed (F = 13.37; df = 4,25; *p* < 0.001). All EPNS treatments resulted in high mortality, whereas the water control showed the lowest mortality.

Overall, the results demonstrate high efficacy of EPNS treatments in microcolonies, with a clear dose-dependent response.

The treatments (bacterial suspension and secondary metabolites) significantly affected colony mortality in the microcolonies, as confirmed by ANOVA.

For larvae, a significant treatment effect was observed (F = 6.30; df = 3,20; *p* < 0.01). The secondary metabolites treatment resulted in the highest mortality (28.3%), differing significantly from the control (0%), whereas the bacterial (18.3%) and LB25% (6.7%) treatments showed intermediate values. Mean mortality ranged from 0.00 (95% CI: 0.00–0.00) to 2.83 (95% CI: 0.40–5.26) dead individuals.

For pupae, a significant treatment effect was also observed (F = 3.56; df = 3,20; *p* < 0.05). The secondary metabolites treatment resulted in the highest mortality (25.0%), differing significantly from the control (0%), whereas the bacterial (16.7%) and LB25% (1.7%) treatments showed intermediate values. Mean mortality ranged from 0.00 (95% CI: 0.00–0.00) to 2.50 (95% CI: 0.13–4.87) dead individuals.

In workers, treatments had a significant effect (F = 4.71; df = 3,20; *p* < 0.05). The secondary metabolites (70.0%) and bacterial (60.0%) treatments showed higher mortality and did not differ significantly from each other, whereas the control showed low mortality (15.0%) and the LB25% treatment showed intermediate values (38.3%). Mean mortality ranged from 1.50 (95% CI: − 0.09–3.09) to 7.00 (95% CI: 3.75–10.3) dead individuals.

For alates, a significant treatment effect was also observed (F = 4.00; df = 3,20; *p* < 0.05). The bacterial treatment resulted in maximum mortality (10%), whereas the secondary metabolites treatment showed high mortality (8.3%), both higher than the control (1.7%). The LB25% treatment showed intermediate values (3.3%). Mean mortality ranged from 0.17 (95% CI: − 0.26–0.59) to 1.00 (95% CI: 1.00–1.00) dead individual.

**Fig. 9:**
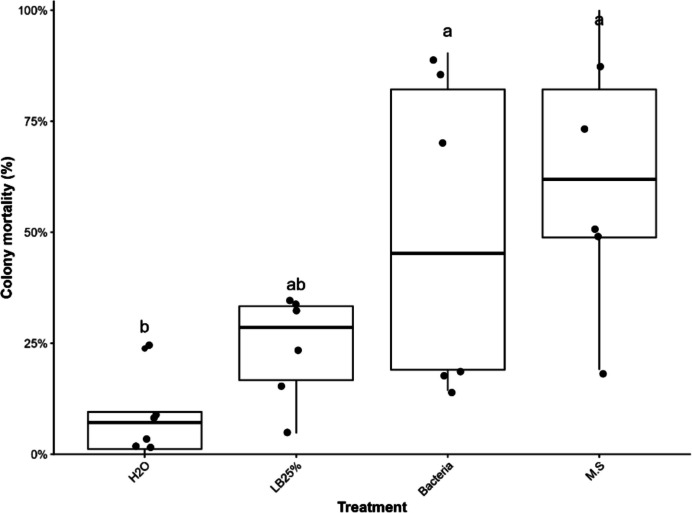
Colony mortality (%) in ant microcolonies subjected to different treatments (H₂O, LB25%, Bacteria, and secondary metabolites [M.S.]) seven days after inoculation. Boxplots represent the distribution of mortality values, with points indicating individual replicates. Different lowercase letters above the boxplots indicate significant differences among treatments according to Tukey’s multiple comparison test (*P* < 0.05)

Overall, the results indicate that both bacterial and secondary metabolite treatments were effective in increasing colony mortality, with effects varying across developmental stages (Fig. 9).

The mortality rates observed in the present study (70–100%, depending on developmental stage and treatment) are comparable to or higher than those reported in previous studies using entomopathogenic nematodes against *Solenopsis* sp. Jouvenaz et al. (1990) conducted a control experiment against *Solenopsis* sp. using entomopathogenic nematodes of the species *Steinernema feltiae*. The results were positive, with mortality reaching up to 58% in queens and approximately 80% in workers and immature stages, using doses of 5,000 and 50,000 infective juveniles (IJs). In this study, intense grooming behavior by workers was observed immediately after treatment application, as well as protective and hygienic behaviors directed toward the immature stages.

Drees et al. (1992) also recorded grooming behavior in red imported fire ants immediately after nematode application in a field experiment. The authors reported that workers vigorously removed nematodes from immature stages, winged forms, and from themselves. In the same study, when evaluating the control of *S. invicta* through the application of *Steinernema carpocapsae* to larvae, pupae, and winged forms under laboratory conditions, mortality ranged from 28 to 100% after 96 h, using doses of 10^3^ and 10^5^ infective juveniles per Petri dish.

Zhang and Han (2011) also observed protective behavior by red imported fire ant workers toward queens in experiments with the nematode *Steinernema carpocapsae*. The authors reported that infected queens accompanied by six workers showed 90% mortality after six to nine days; however, when accompanied by 100 workers, queen mortality was reduced to 40% after nine days.

The lower mortality observed in workers in the present study, both in assays with isolated stages and in microcolonies, may be associated with grooming behavior and social immunity mechanisms. It is well known that workers perform intense self-grooming and allogrooming (Cremer et al. 2007), which may reduce pathogen load through the mechanical removal of infective juveniles from the body surface before successful penetration, as well as limit its spread within the colony.

In an experiment conducted by Zarzuela et al. (2012), using the nematodes *Steinernema carpocapsae* and *Heterorhabditis* sp. for the control of *Monomorium floricola* (Jerdon, 1851) (Hymenoptera: Formicidae), approximately 2,000 to 3,000 ants, including workers, larvae, and queens, were maintained in Petri dishes (5 cm in diameter × 1 cm in depth), with lids painted black to reduce light intensity. Approximately 500 workers died following nematode application; however, none of the workers showed signs of infection, in contrast to the immature stages.

The authors also reported that colony size did not decrease throughout the experiment and, in some cases, even increased in the number of individuals. This population increase may reflect a compensatory mechanism, representing an attempt by the colony to recover under control pressure.

Microcolony assays more closely resemble the conditions likely to be encountered under field situations, particularly with respect to colony organization and worker-mediated protective behaviors. However, future studies will be necessary to evaluate the persistence of entomopathogenic nematodes in the soil, since their survival and infectivity depend on suitable soil conditions, including moisture, temperature, and other physicochemical characteristics (Rohde et al. 2010). In addition, the behavior of fire ants, which may relocate the colony in response to disturbance (CABI 2025), represents an important challenge for field application. Therefore, the application method must be carefully designed to allow silent and unobtrusive delivery, enabling the biological agent to reach the colony without triggering defensive or migratory responses. Furthermore, the development of suitable formulations that preserve nematode viability and facilitate field administration will be essential for practical use.

In the present study, survival over time was not recorded, which prevented the application of time-to-event analyses such as Kaplan–Meier survival curves or Cox proportional hazards models. As a result, differences in the dynamics of mortality among treatments could not be assessed. In addition, although grooming and removal behaviors were qualitatively observed during the experiments, these behaviors were not quantified. Therefore, it was not possible to establish a direct relationship between social immunity mechanisms and the observed differences in susceptibility among castes, particularly in workers. Future studies should incorporate temporal mortality data and quantitative behavioral observations to better understand the role of social defenses and their interaction with entomopathogenic agents at the colony level.

### Identification of collected specimens

The analysis of gene sequences from samples collected from the nests used in the experiments showed high similarity with the species *Solenopsis invicta* (Table 2). In a study conducted at the Instituto Biológico in 2010, haplotypes showing sequence similarity to both *S. saevissima* and *S. invicta* were identified; however, the predominance of *S. invicta* in the environment was observed in 80% of the sampled nests (Gusmão et al. 2010). In the municipality of Rio Claro, São Paulo State, nests of *S. invicta* were identified with an abundance of 92.3% of the total nests when compared to *S. saevissima* (Fox, Solis & Bueno, 2008).

**Table 2:** Identification of specimens collected for the experiments, respective GenBank accession numbers, haplotypes, and percentage similarity according to BOLD Systems and BLAST

Sample code	Species	Similarity (BLAST)	DNA haplotype	GenBank accession number
V1	*Solenospsis invicta*	*99,27%*	H58	AY950752.1
V2	*Solenospsis invicta*	*99,03%*	H42	AY950737.1
V3	*Solenospsis invicta*	*99,27%*	H58	*AY950752.1*
L1	*Solenospsis invicta*	*100%*	H41	AY950736.1
L2	*Solenospsis invicta*	*99,27%*	H58	*AY950752.1*
L5	*Solenospsis invicta*	*99,03%*	H42	AY950737.1
A1	*Solenospsis invicta*	*98,79%*	H58	*AY950752.1*
A2	*Solenospsis invicta*	*98,31%*	H42	AY950737.1
A3	*Solenospsis invicta*	*98,55%*	H58	*AY950752.1*
B1	*Solenospsis invicta*	*99,75%*	H41	AY950736.1
R2	*Solenospsis invicta*	*98,78%*	H58	*AY950752.1*
NA	*Solenospsis invicta*	*99,51%*	H58	*AY950752.1*

## Conclusions

In laboratory assays, entomopathogenic nematodes at a concentration of 300 IJs/insect caused high mortality, ranging from 70 to 100% across all developmental stages of fire ants (*Solenopsis invicta*), including larvae, pupae, workers, and winged forms. Among the evaluated stages, larvae and winged forms were the most susceptible to both the bacterium *Xenorhabdus szentirmaii* and its secondary metabolites, a pattern consistently observed in assays with isolated stages and in microcolony experiments. These findings indicate the strong potential of both entomopathogenic nematodes and bacterial-derived bioactive compounds as biological control agents against *S. invicta*. Furthermore, the comparable efficacy observed between the bacterial suspension and its secondary metabolites suggests that these compounds may contribute substantially to the observed mortality. Future studies under semi-field and field conditions are needed to confirm their effectiveness and evaluate their potential for incorporation into integrated pest management programs.

## Supplementary Information

Below is the link to the electronic supplementary material.

Supplementary file1 (DOCX 16 KB)

## Data Availability

The data that support the findings of this study are available from the corresponding author upon reasonable requestand.
