# Supplementary material for: Entomopathogenic Nematode Steinernema rarum and its Symbiotic Bacterium against Fire Ants (Solenopsis sp.) under Laboratory Conditions
Source: Neotrop Entomol. 2026 May 21;55(1):53. doi: 10.1007/s13744-026-01400-y (PMC13194329; doi:10.1007/s13744-026-01400-y)
Supplement: Supplementary file 1 — Supplementary file1 (DOCX 16 KB) [file 13744_2026_1400_MOESM1_ESM.docx]

**Statistical analysis: EPNs – developmental stages**

Treatments significantly affected mortality across all evaluated developmental stages. For larvae, a strong treatment effect was observed (ANOVA: F(4, 40) = 58.03; p = 4.01 × 10⁻¹⁶; η² = 0.85; 95% CI: 0.78–1.00). All EPN treatments resulted in high mortality rates (86.7–100%), with no significant differences among them, and all were significantly higher than the control (H₂O: 8.9%). Mean mortality ranged from 8.67 (95% CI: 7.73–9.61) to 10.0 (95% CI: 10.0–10.0) dead individuals.

For pupae, a significant treatment effect was also detected (F(4, 40) = 121.10; p < 2 × 10⁻¹⁶; η² = 0.92; 95% CI: 0.88–1.00). The EPN_50, EPN_150, and EPN_300 treatments resulted in the highest mortality rates (83.3–88.9%), differing significantly from EPN_5 (61.1%) and the control (3.3%). The EPN_5 treatment showed intermediate values (95% CI: 4.29–7.93).

In workers, treatments also had a significant effect (F(4, 40) = 34.03; p = 2.19 × 10⁻¹²; η² = 0.77; 95% CI: 0.66–1.00). The EPN_150 (77.8%; 95% CI: 5.98–9.57) and EPN_300 (85.6%; 95% CI: 6.75–10.4) treatments resulted in higher mortality, differing significantly from the other treatments. In contrast, EPN_5 (24.4%), EPN_50 (21.1%), and the control (7.8%) did not differ from one another, indicating lower efficacy at these concentration levels.

For alates, a significant treatment effect was likewise observed (F(4, 40) = 47.80; p = 1.01 × 10⁻¹⁴; η² = 0.83; 95% CI: 0.74–1.00). The EPN_50, EPN_150, and EPN_300 treatments produced high mortality rates (83.3–92.2%), whereas EPN_5 showed an intermediate effect (57.8%; 95% CI: 4.30–7.26), and the control showed low mortality (16.7%; 95% CI: 1.28–2.05).

Effect sizes were high across all stages (η² = 0.77–0.92), indicating a strong influence of treatments on mortality. Differences between treatments and the control were biologically meaningful, reaching more than 90 percentage points in larvae.

Dose–response analysis revealed variation in susceptibility among developmental stages. Larvae showed the lowest LD₅₀ values (0.05; 95% CI: −0.16–0.27), indicating high sensitivity to the treatments, whereas workers showed greater tolerance (LD₅₀ = 89.93; 95% CI: 66.32–113.53). LD₉₀ values were higher and presented wide confidence intervals, especially for pupae and alates, suggesting greater variability in response during these stages.

**Statistical analysis: Bacteria – developmental stages**

Treatments significantly affected mortality across different developmental stages of the ants. For larvae, a strong treatment effect was observed (ANOVA: F(3, 32) = 42.60; p = 2.78 × 10⁻¹¹; η² = 0.80). Treatments with bacteria (75.6%) and secondary metabolites (85.6%) resulted in the highest mortality rates and did not differ significantly from each other, but were significantly higher than the control (H₂O: 8.9%) and the LB25% treatment (16.7%). Mean mortality ranged from 7.56 (95% CI: 6.68–8.43) to 8.56 (95% CI: 7.47–9.64) dead individuals.

For pupae, a significant effect was also detected (F(3, 32) = 4.98; p = 0.00599; η² = 0.32). The bacterial treatment resulted in the highest mortality (28.9%), differing significantly from the control (3.3%) and LB25% (4.4%), whereas the secondary metabolites treatment showed intermediate values (8.9%). Mean mortality ranged from 0.33 (95% CI: −0.05–0.72) to 2.89 (95% CI: 1.66–4.11) dead individuals.

In workers, treatments had a significant effect (F(3, 32) = 7.34; p = 0.000705; η² = 0.41). The bacteria (71.1%), secondary metabolites (62.2%), and LB25% (61.1%) treatments showed similarly high mortality and did not differ significantly from one another, all being superior to the control (7.8%). Mean mortality ranged from 0.78 (95% CI: −0.06–1.62) to 7.11 (95% CI: 5.33–8.89) dead individuals.

For alates, no significant treatment effect was observed (F(3, 32) = 1.40; p = 0.262; η² = 0.12). Mean mortality ranged from 1.67 (95% CI: 1.28–2.05) to 3.00 dead individuals, with no significant differences among treatments.

Overall, the results demonstrate that treatments were highly effective in larvae, had a moderate effect in pupae and workers, and were ineffective in alates. The similarity between the bacterial and secondary metabolite treatments suggests that bioactive compounds produced by the bacteria play an important role in the observed mortality.

**Statistical analysis: microcolony – EPNs**

Treatments significantly affected mortality across all evaluated developmental stages in the microcolonies. For larvae, a strong treatment effect was observed (ANOVA: F(4, 25) = 25.00; p = 2.01 × 10⁻⁸; η² = 0.80; 95% CI: 0.66–1.00). The EPN_50, EPN_150, and EPN_300 treatments showed the highest mortality, with means ranging from 4.67 (95% CI: 3.81–5.52) to 4.83 (95% CI: 4.40–5.26) dead individuals, whereas the control showed no mortality.

For pupae, a significant treatment effect was also detected (F(4, 25) = 17.96; p = 4.58 × 10⁻⁷; η² = 0.74; 95% CI: 0.56–1.00). The EPN_50, EPN_150, and EPN_300 treatments resulted in higher mortality, ranging from 3.33 (95% CI: 2.25–4.42) to 3.83 (95% CI: 1.69–5.98) dead individuals, whereas the control showed no mortality.

In workers, treatments had a significant effect (F(4, 25) = 11.94; p = 1.46 × 10⁻⁵; η² = 0.66; 95% CI: 0.42–1.00). The EPN_150 (7.67; 95% CI: 5.83–9.50), EPN_300 (8.00; 95% CI: 5.26–10.7), and EPN_50 (7.00; 95% CI: 4.61–9.39) treatments showed higher mortality, whereas the control showed low mortality (1.50; 95% CI: −0.09–3.09).

For alates, a significant treatment effect was also observed (F(4, 25) = 13.37; p = 5.85 × 10⁻⁶; η² = 0.68; 95% CI: 0.46–1.00). All EPN treatments resulted in maximum mortality (mean = 1.00), whereas the control showed reduced mortality (0.17; 95% CI: −0.26–0.59).

Overall, effect sizes were high across all stages (η² = 0.66–0.80), indicating a strong influence of treatments on mortality. These results demonstrate high treatment efficacy in microcolonies, with a consistent response across the different developmental stages.

**Statistical analysis: microcolony – bacteria**

Treatments significantly affected mortality across all evaluated developmental stages in the microcolonies. For larvae, a significant treatment effect was observed (ANOVA: F(3, 20) = 6.30; p = 0.00349; η² = 0.49; 95% CI: 0.16–1.00). The secondary metabolites treatment resulted in the highest mortality (28.3%), differing significantly from the control (0%), whereas the bacterial (18.3%) and LB25% (6.7%) treatments showed intermediate values. Mean mortality ranged from 0.00 (95% CI: 0.00–0.00) to 2.83 (95% CI: 0.40–5.26) dead individuals.

For pupae, a significant treatment effect was also observed (F(3, 20) = 3.56; p = 0.0325; η² = 0.35; 95% CI: 0.02–1.00). The secondary metabolites treatment resulted in the highest mortality (25.0%), differing significantly from the control (0%), whereas the bacterial (16.7%) and LB25% (1.7%) treatments showed intermediate values. Mean mortality ranged from 0.00 (95% CI: 0.00–0.00) to 2.50 (95% CI: 0.13–4.87) dead individuals.

In workers, treatments had a significant effect (F(3, 20) = 4.71; p = 0.0121; η² = 0.41; 95% CI: 0.08–1.00). The secondary metabolites (70.0%) and bacterial (60.0%) treatments showed higher mortality and did not differ significantly from each other, whereas the control showed low mortality (15.0%) and the LB25% treatment showed intermediate values (38.3%). Mean mortality ranged from 1.50 (95% CI: −0.09–3.09) to 7.00 (95% CI: 3.75–10.3) dead individuals.

For alates, a significant treatment effect was also observed (F(3, 20) = 4.00; p = 0.0222; η² = 0.37; 95% CI: 0.04–1.00). The bacterial treatment resulted in maximum mortality (10%), whereas the secondary metabolites treatment showed high mortality (8.3%), both higher than the control (1.7%). The LB25% treatment showed intermediate values (3.3%). Mean mortality ranged from 0.17 (95% CI: −0.26–0.59) to 1.00 (95% CI: 1.00–1.00) dead individual.

Overall, effect sizes were moderate (η² = 0.35–0.49), indicating a consistent influence of treatments on mortality. These results demonstrate that secondary metabolites showed efficacy comparable to or greater than that of the bacteria across different developmental stages, particularly in larvae and pupae.
